# Hyperuricemia Is Associated With the Risk of Atrial Fibrillation Independent of Sex: A Dose-Response Meta-Analysis

**DOI:** 10.3389/fcvm.2022.865036

**Published:** 2022-04-07

**Authors:** Jianhua Xiong, Wen Shao, Peng Yu, Jianyong Ma, Menglu Liu, Shan Huang, Xiao Liu, Kaibo Mei

**Affiliations:** ^1^Department of Cardiology, The Affiliated Hospital of Jiangxi University of Chinese Medicine, Nanchang, China; ^2^Key Laboratory of Cardiovascular Diseases in Chinese Medicine, Nanchang, China; ^3^Department of Endocrine, The Second Affiliated Hospital of Nanchang University, Nanchang, China; ^4^Department of Pharmacology and Systems Physiology, University of Cincinnati College of Medicine, Cincinnati, OH, United States; ^5^Department of Cardiology, The Seventh Hospital of Zhengzhou, Zhengzhou, China; ^6^Department of Psychiatry, The Third People's Hospital of Gan Zhou, Ganzhou, China; ^7^Department of Cardiology, Sun Yat-sen Memorial Hospital of Sun Yat-sen University, Guangzhou, China; ^8^Guangdong Province Key Laboratory of Arrhythmia and Electrophysiology, Guangzhou, China; ^9^Department of Anesthesia, The People's Hospital of Shangrao, Shangrao, China

**Keywords:** atrial fibrillation, serum uric acid, sex difference, meta-analysis, hyperuricemia

## Abstract

**Background:**

Conflicting findings of the association between serum uric acid (SUA) and atrial fibrillation (AF) have been reported in both men and women. The sex-specific associations between SUA and the risk of AF are unclear, although hyperuricemia is independently associated with the risk of AF. We performed this meta-analysis to assess the sex-specific effect of SUA on the risk of AF.

**Methods:**

The PubMed, EMBASE, and Cochrane Library databases were searched up to October 3, 2021, for studies that reported sex-specific associations of SUA levels with AF. Linear relationships were assessed by the generalized least squares trend estimation. This study was registered with PROSPERO (42020193013).

**Results:**

Ten eligible studies with 814,804 participants (415,779 men and 399,025 women) were identified. In the category analysis, high SUA was associated with an increased risk of AF in both men (OR: 1.42; 95% CI, 1.18–1.71, I^2^ = 34%) and women (OR: 2.02; 95% CI, 1.29–3.16, I^2^ = 70%). In the dose-response analysis, for each 60 μmol/L (1 mg/dL) increase in the SUA level, the risk of AF increased by 15% (OR: 1.15; 95% CI, 1.07–1.25, I^2^ = 74%) in men and 35% (OR: 1.35; 95% CI, 1.18–1.53, I^2^ = 73%) in women. There was a borderline difference in the impact of SUA on the risk of AF between men and women (*P* for interaction = 0.05). A significant linear relationship between SUA and the risk of AF was observed in men (*P* for non-linearity = 0.91) and women (*P* for non-linearity = 0.92).

**Conclusions:**

This study suggested that there was a significant linear relationship between SUA and the risk of AF among men and women, with a higher risk estimate for women. Additional trials are required to assess the effect of reduced SUA therapy on AF incidence.

**Systematic Review Registration:**

https:www.crd.york.ac.uk/PROSPERO/, identifier: CRD 42020193013.

## Introduction

Atrial fibrillation (AF) is the most common sustained cardiac arrhythmia and it is associated with an increased risk of stroke, thromboembolic events, heart failure and other comorbidities. Due to its high rate of disability and mortality, AF has become a worldwide health problem (1). It is well known that sex is an independent risk factor for AF development. Accumulating evidence indicates that AF affects men and women differently in all aspects, including clinical presentation, prognosis, and outcomes (2, 3). Some studies have suggested that women with AF had more comorbidities, were more often at the highest risk for stroke, and were more often symptomatic than men (4–6). Recently, a meta-analysis of 30 studies involving more than four million participants showed that the risk of cardiovascular disease and death associated with AF was twice as high in women compared with men (7).

The specific pathophysiological mechanisms of AF are not yet fully understood but are considered complex and multifactorial (8). Neurohormone activation, oxidative stress upregulation and immune activation jointly promote the occurrence of AF (9–11). Serum uric acid (SUA), as the final purine metabolite catalyzed by xanthine oxidoreductase (XO), reflects the degree of oxidative stress *in vivo* (12). Over the past two decades, numerous epidemiological studies have shown that elevated SUA levels are closely related to various diseases, including hypertension (13), coronary heart disease (14), cardiovascular disease (15, 16), obesity (17), diabetes (18), metabolic syndrome (19), liver dysfunction (20), and chronic kidney disease (21). Moreover, a variety of studies have demonstrated that a high SUA level is significantly associated with AF and may be a potential risk factor for AF (22–28).

SUA levels are affected by sex, and SUA levels in men are higher than those in women in general, although their SUA levels increase after menopause. More recently, several observational studies have evaluated sex-specific associations of SUA with incident AF, but the results were contradictory (29–38). For instance, the Scottish Heart Health Extended Cohort (SHHEC) study proposed that elevated SUA was associated more strongly with AF in women than in men (37), while the Atherosclerosis Risk in Communities (ARIC) study demonstrated that an independent association with SUA was only observed in women after adjusting for other cardiovascular risk factors (29). Considering that the potential sex-specific correlation between elevated SUA levels and AF would provide new insights into the etiology of AF, studies that have probed this issue and synthesized the conflicting evidence are definitely needed (39). However, no such studies have been carried out, and all previous meta-analyses only focused on the relationship between SUA and AF, rather than the sex-specific association (26–28).

Therefore, we performed a meta-analysis to evaluate whether there are sex-related differences in the association of SUA levels with the risk of AF.

## Methods

### Protocol Registration and Search Strategy

We registered the protocol in PROSPERO (International prospective register of systematic reviews https:www.crd.york.ac.uk/PROSPERO/-registration number-CRD 42020193013). This meta-analysis was performed in accordance with the PRISMA statement (Preferred Reporting Items for Systematic Reviews and Meta-Analyses) (Supplementary Table S1) (40).

Database search, literature selection, quality assessment, and data extraction were carried out independently by two authors (J-H. X and W. S). Any disagreements were settled through discussions. The PubMed, EMBASE, and Cochrane Library databases were searched up to October 3, 2021 for all related studies. No restrictions were imposed on the language of the publications. The following MeSH words were used in our search strategies: (“*uric acid”* OR “*urate”* OR “*hyperuricemia”* OR “*gout”*) AND (“*atrial fibrillation” OR “atrial flutter” OR “atrial tachycardias”*).

### Selection Criteria and Study Selection

The inclusion criteria were as follows: (1) reported sex-specific association of SUA levels with AF or reported for a specific sex; (2) risk estimates [risk ratio (RR), hazard ratio (HR), or odds ratio (OR)] and corresponding 95% CIs of the association between SUA and AF were reported. In addition, studies were excluded if they met the following conditions: (1) reviews, meta-analyses, congress abstracts, practice guidelines, patents, cases, editorials, replies, or comments; (2) the required data were not extractable even after contacting the corresponding authors for further information.

### Data Collection and Quality Assessment

The data were collected into a predesigned standard spreadsheet. The following data were abstracted from each included study: study characteristics (first author's name, publication year, location, and study design), patient characteristics (type of population, sample size, age, percentage of men, follow-up duration and AF detection), and outcomes (number of events, adjusted OR/RRs and the corresponding 95% CI, and adjustments).

We used the Joanna Briggs Institute critical appraisal checklist to evaluate the quality of the cross-sectional studies (41). The Newcastle–Ottawa Scale (NOS) was applied to assess the quality of the case-control and cohort studies (42). The NOS scoring criteria included three aspects: (1) the selection of the subjects; (2) the comparability of the subjects; and (3) the clinical outcome or exposure. The total NOS score was 9, with a score of 7 or more considered high quality.

### Statistical Analysis

To examine the relationships between SUA level and the risk of AF among men and women, we pooled the ORs and the corresponding 95% CIs by using the inverse-variance method. The RRs and HRs were considered equivalent to ORs. For the category analysis, both patients with highest vs. lowest and hyperuricemia vs. normal was analyzed. Hyperuricemia was defined as a SUA level > 7 mg/dl in three studies (22, 25, 31) for men and >5.7 mg/dl in two studies (22, 31) for women.

We performed dose-response analysis and calculated study-specific slopes (linear trends) by using the method proposed by Greenland and Longnecker (43). Additionally, 95% CIs were calculated from the natural logs of the ORs and CIs across the SUA categories. In addition, we assessed the potential non-linear association between SUA and the risk of AF by using restricted cubic splines with 3 knots (44, 45). Heterogeneity among the included studies was evaluated by the χ^2^ test (with a *P*-value < 0.10 considered significant) and *I*^2^ test (25%, 50%, and 75% represent low, moderate and high heterogeneity, respectively) (46). ORs and the corresponding 95% CIs were pooled by using the random-effects model, considering the potential heterogeneity across studies.

Subgroup and meta-regression analyses were used to identify potential sources of heterogeneity among the analyses. Studies were stratified by the mean age of the participants; region; NOS scores; study design; sample size; population; and adjustments for important risk factors, including age, body mass index (BMI), smoking, history of hypertension, and diabetes. We performed a sensitivity analysis by omitting 1 study at a time to assess the robustness of the conclusions. The possibility of publication bias was assessed by funnel plots, Egger's test (47), and Begg's test (48). All analyses were conducted by using Stata 14.0 (Stata Corp LP, College Station, TX, USA) and Review Manager (RevMan) version 5.3 (The Cochrane Collaboration 2014; Nordic Cochrane Centre Copenhagen, Denmark). A *p*-value < 0.05 was considered significant.

## Results

### Literature Search

**Figure 1:**
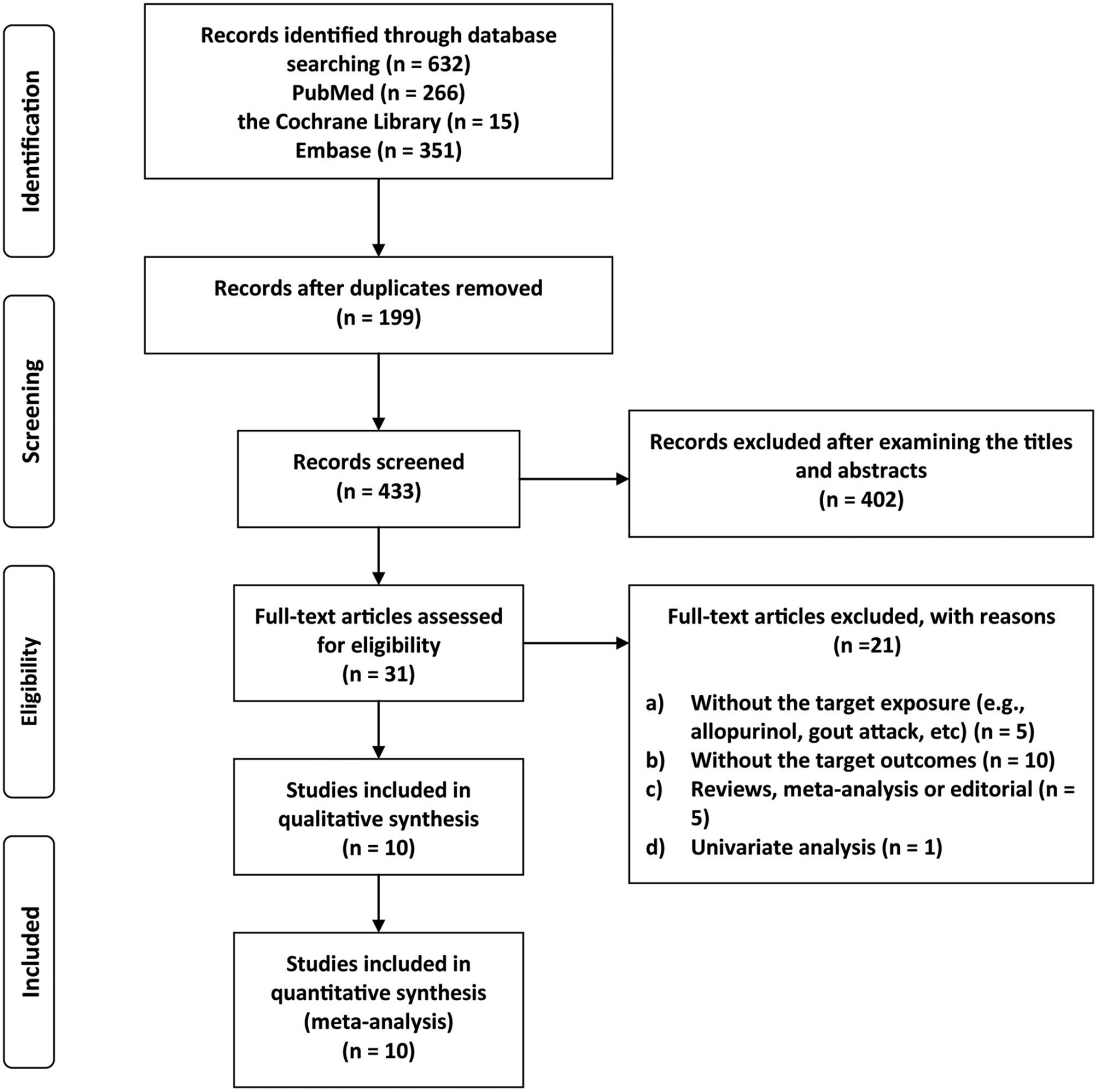
Flow chart of study selection.

The literature screening process is shown in Figure 1. The initial database search identified 632 records, and after screening the titles and abstracts, 31 articles remained for full text screening. Among the 31 articles selected for full-text review, we included 10 studies (29–38) and excluded 21 for the following reasons: (1) the studies did not report the target exposure (e.g., allopurinol, gout attack, etc., *n* = 5); (2) the studies did not report sex-specific outcomes (*n* = 10); (3) editorials or review articles (*n* = 5); and (4) univariate analysis (*n* = 1). The reasons for exclusion are listed in Supplementary Table S2.

### Study Characteristics and Study Quality

The basic characteristics of all included studies are summarized in Table 1. Overall, 10 studies were included (29–38), with 814,804 participants (415,779 men and 399,025 women). The populations included in the studies ranged from 400 to 353,613 for the sample size, 4–22.6 years for follow-up, and 38–64 years for the mean or median age. Two studies were designed as cross-sectional (33, 34), 3 were retrospective cohorts (32, 36, 38), and 5 were prospective cohorts (29–31, 35, 37). Most studies were well adjusted for potential confounders. Six reports were from Asia (China = 3, Japan = 2, and Korean = 1) (32–36, 38), 3 from Europe (Italy = 1, Scotland = 1 and Norway = 1) (30, 31, 37), and 1 from the USA (29). In accordance with the Joanna Briggs Institute Critical Appraisal Checklist, 2 cross-sectional studies met all nine criteria (33, 34). which meant that these articles applied rigorous methodology (Supplementary Table S3). Based on the NOS, the quality of all of the included reports was high (29–32, 35–38), with a score range of 7–9 (Supplementary Table S4).

**Table 1:** Basic characteristics of the articles included in the meta-analysis of serum uric acid and risk of atrial fibrillation in men and women.

**References**	**Location**	**Design**	**Population**	**Sample size**	**Follow-up time (years)**	**Age (years)**	**AF detection**	**Adjustments for confounders**
Tamariz et al. (29)	USA	Prospective cohort	General population	Men: 7,054 Women: 8,328	16	54	ECG	Age, race, education, field center, BMI, alcohol, serum glucose, SBP, DBP, and LDL-cholesterol, CHD and HF, diuretic use, P-wave duration, MI and HF
Valbusa et al. (30)	Italy	Prospective cohort	Type2 DM	Men: 235 Women: 165	10	64	ECG	Age, gender, BMI, SBP, hypertension treatment, electrocardiographic PR interval, and history of HF
Nyrnes et al. (31)	Norway	Prospective cohort	General population	Men: 3,090 Women: 3,218	10.8	61	ECG	Age, sex, smoking, alcohol, PA, SBP, eGFR, BMI, total cholesterol, HDL-cholesterol, CHD, DM, anti-hypertensive/diuretic/ACEI treatment, and MI incidence
Ding et al. (32)	China	Retrospective cohort	DM patients	Men: 7,449 Women: 1,601	NA	57.2	ECG	Age, BMI, SBP, DBP, FBG, TG, TC, LDL, UA, BUN, CRP, PP, smoking, history of MI and anti-hypertensive medication
Sun et al. (33)	China	Cross-sectional	General population	Men: 5,170 Women: 6,168	NA	53.8	ECG	Age, BMI, waist circumference, SBP, DBP, fasting blood glucose, TC, TG, smoking, drinking, MI, low LVEF, LV hypertrophy, and family history of AF
Chen et al. (34)	China	Cross-sectional	General population	Men: 4,686 Women: 4,251	NA	42.1	ECG	Age, smoking, alcohol use, diuretics use, statins use, hypertension, DM, MI, TIA/stroke, dyslipidemia, HF and gout
Kwon et al. (36)	Korea	Retrospective cohort	General population	Men: 161,362 Women: 121,111	5.4	38	ECG	Age, sex, BMI, smoking status, alcohol intake, regular exercise, medical history hypertension, DM, stroke, CAD, SBP and CKD
Kawasoe et al. (35)	Japan	Prospective cohort	General population	Men: 53,416 Women: 58,150	4.1	53.8	ECG	Age, BMI, Scr, smoking and drinking status, and presence of hypertension, DM, and dyslipidemia
Peters et al. (37)	Scotland	Prospective cohort	General population	Men: 7,552 Women: 8,185	22.6	49	ECG	Age, family history of CHD, socioeconomic status, cigarette equivalent dose, systolic BP, TC, and HDL
Seki et al. (38)	Japan	Retrospective cohort	General population	Men: 165,765 Women: 187,848	3.2	40	ICD	Age, overweight/obesity, high waist circumference, Hypertension, DM mellitus, dyslipidemia, CKD, cigarette smoking, alcohol drinking, and PA

*AF, atrial fibrillation; BMI, body mass index; DM, diabetes mellitus; CKD, chronic kidney disease; CAD, coronary artery disease; BP, blood pressure; SBP, systolic blood pressure; DBP, diastolic blood pressure; PP, pulse pressure; PA, physical activity; eGFR, estimated glomerular filtration rate; CHD, coronary heart disease; MI, myocardial infarction; HF, heart failure; ACEI, angiotensin converting enzyme inhibitor; HDL, high-density lipoprotein; LDL, low-density lipoprotein; Scr, serum creatinine; TIA, transient ischemic attack; TC, total cholesterol; TG, triglyceride; LVEF, left ventricular ejection fraction; LV, left ventricular; UA, serum uric acid; FBG, fasting blood glucose; BUN, blood urea nitrogen; CRP, systemic inflammation; NA, not available; ECG, electrocardiogram; ICD, implantable cardioverter defibrillator*.

### SUA Levels and Risk of AF in Men and Women

#### Categorical Analysis of SUA and AF

**Figure 2:**
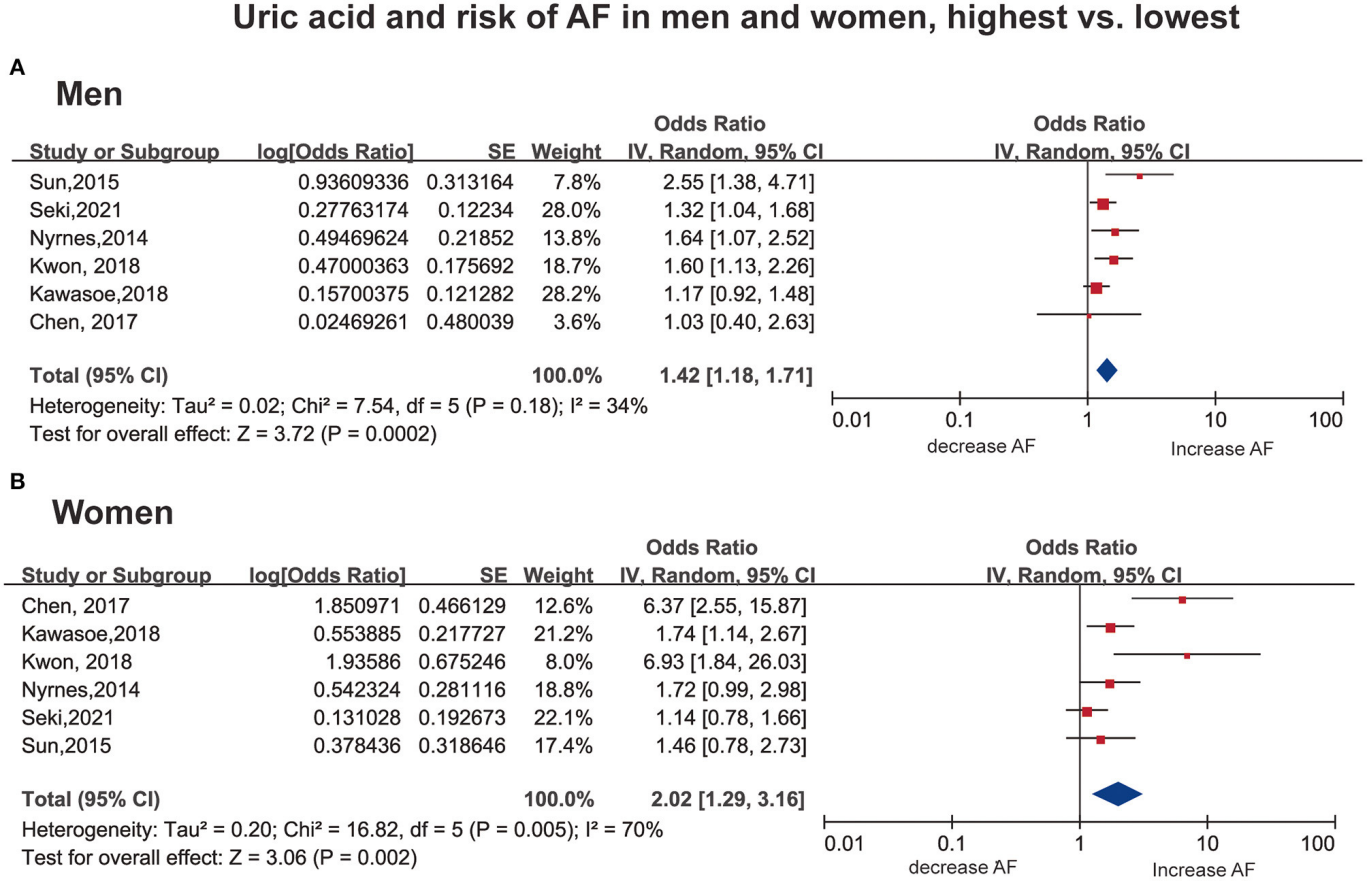
Forest plot for the risk of AF with the highest vs. the lowest serum uric acid among men **(A)** and women **(B)**. AF, atrial fibrillation SE, standard error.

Six studies (31, 33–36, 38) with 393,489 men/380,746 women reported the association as a category variable between the SUA levels and AF. In the analyses of highest-vs.-lowest, an increased risk of AF with higher SUA levels was found (OR: 1.42; 95% CI, 1.18–1.71) in men, with low evidence of heterogeneity (I^2^ = 34%, Figure 2A). The heterogeneity was reduced to 0% when Sun et al. (33) was excluded, with a similar result (OR: 1.33; 95% CI, 1.15–1.53, I^2^ = 0%).

In women, a consistent result was observed (OR: 2.02; 95% CI, 1.29–3.16), with high heterogeneity (I^2^ = 70%, Figure 2B). Notably, two studies (34, 36) showed an OR over 6, which is much larger than the others. The results remained significant with no evidence of heterogeneity (OR: 1.44; 95% CI, 1.14–1.82, I^2^ = 0%) when excluding the above studies (34, 36).

Only three studies (33, 34, 36) examined the gender-specific association between hyperuricemia vs. the normal SUA and AF. The results showed that patients with hyperuricemia were associated with increased risk of AF in men (OR: 1.71; 95% CI, 1.16–2.54, I^2^ = 31%) but not in women (OR: 2.93; 95% CI, 0.69–12.38, I^2^ = 85%) (Supplementary Figure S1).

#### Dose-Response Association Between SUA and the Risk of AF in Men and Women

**Figure 3:**
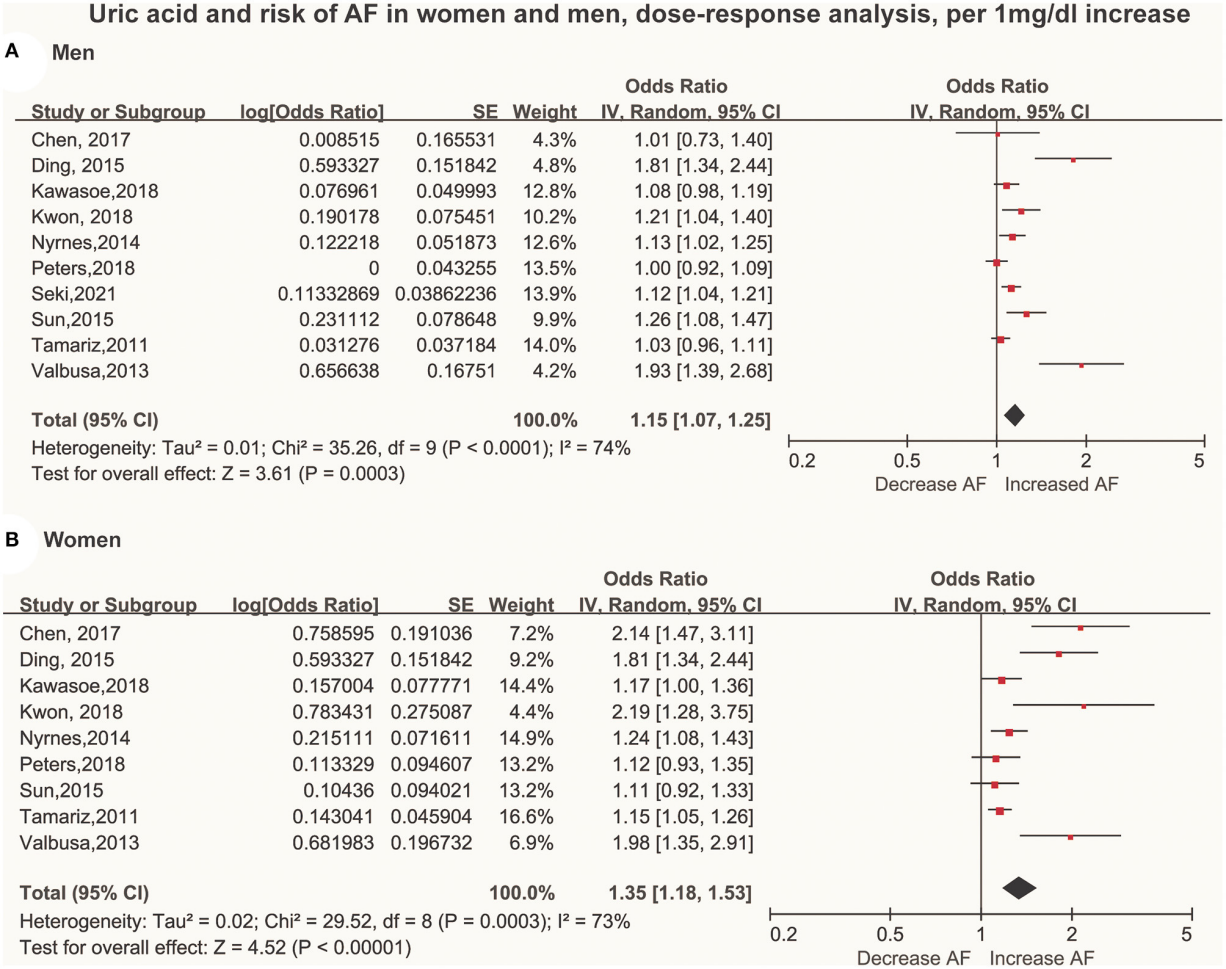
Forest plot of the dose-response association between serum uric acid levels and AF among men **(A)** and women **(B)**. Serum uric acid was analyzed as per a 60 μmol/L (1 mg/dL) increase. AF, atrial fibrillation SE, standard error.

**Figure 4:**
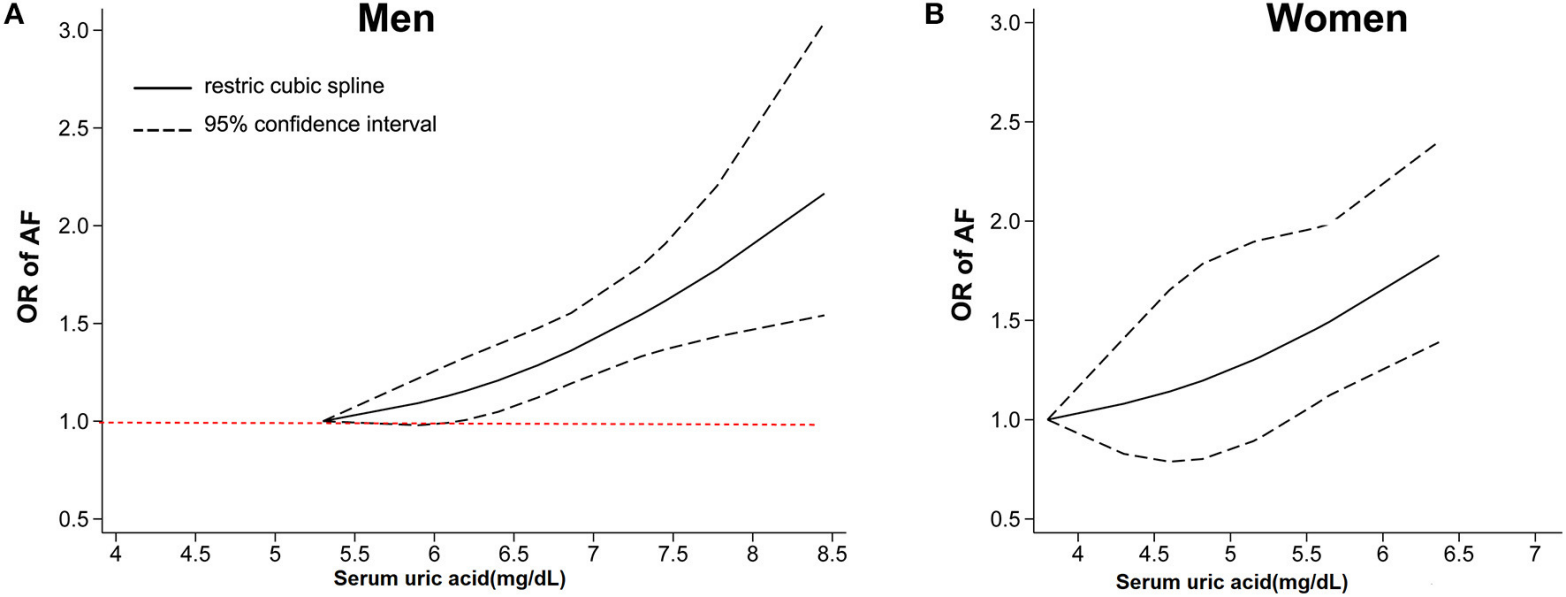
Dose-response analysis between serum uric acid levels and the risk of AF among men **(A)** and women **(B)**. The dose-response association was fitted by the restricted cubic splines model, and the solid and dashed lines represent the estimated odds ratio and the 95% confidence interval, respectively. AF, atrial fibrillation OR, odds ratio.

A total of 10 articles (29–38) with 415,779 men and 211,177 women were included in the dose-response analysis. Ten articles (29–38) with 415,779 participants reported an association between the SUA levels and AF in men. The results showed that each 60 μmol/L (1 mg/dL) increment in SUA increased the risk of AF by 15% (OR: 1.15; 95% CI, 1.07-1.25) in men, with significant heterogeneity (I^2^ = 74%; Figure 3A). There was no evidence of a non-linear association between the SUA levels and the AF risk (*P* for non-linearity = 0.91; Figure 4A).

Nine studies (29–37) with 211,177 participants reported an association between the SUA levels and AF in women. The results showed that each 60 μmol/L (1 mg/dL) increment in SUA increased the risk of AF by 35% (OR: 1.35; 95% CI, 1.18–1.53) in women, with high heterogeneity (I^2^ = 73%; Figure 3B). There was no evidence of a non-linear association between the SUA levels and the AF risk (*P* for non-linearity = 0.92; Figure 4B).

There was a borderline significant difference in the impact of SUA [per 60 μmol/L (1 mg/dL) increase] on the risk of AF between men and women (*P* for interaction = 0.05).

#### Publication Bias

As shown in Supplementary Figure S2, possible publication bias was detected by funnel plots, Egger's and Begg's tests. Thus, we used a trim-and-fill method to adjust for publication bias. However, the results did not change after applying the trim-and-fill test, indicating that the impact of this bias was likely insignificant and that our results were credible (Supplementary Figure S3).

#### Subgroup, Meta-Regression, and Sensitivity Analyses

We conducted a subgroup analysis by patient characteristics, such as age, region, and confounding factors. As shown in Table 2, a positive association between SUA and the risk of AF among men and women persisted in almost all subgroup analyses defined by sex, region, sample size, study quality and adjustment for clinical confounding, and there was little evidence of heterogeneity among any of these subgroups with meta-regression analyses. There was a stronger association between SUA and the risk of AF among reports with diabetic populations, whether in men (*P* heterogeneity <0.001) or women (*P* heterogeneity = 0.001). Sensitivity analyses performed by omitting each study did not change the conclusion, which supported the robustness of our results (Data not shown). Further sensitivity analysis performed by removing the only study (38) that used an implantable cardioverter-defibrillator (ICD) to detect AF showed similar results (Supplementary Table S5).

**Table 2:** Subgroup analysis of serum uric acid and risk of atrial fibrillation in men and women, per 1 mg/dL increment.

		**Men**					**Women**				
**Items**		**Number of studies**	**OR**	**95% CI**	**I^2^**	**Within subgroup**	**Number of studies**	**OR**	**95% CI**	**I^2^**	**Within subgroup**
Age	<65 years	10	1.15	1.07–1.25	74%	NA	9	1.35	1.18–1.53	73%	NA
	≥65 years	0	NA	NA	NA		0	NA	NA	NA	
Region	Northern America	1	1.01	0.73–1.40	NA	0.49	1	1.15	1.05–1.26	NA	0.13
	Europe	3	1.28	1.01–1.63	79%		3	1.27	0.99–1.63	85%	
	Asia	6	1.19	1.08–1.31	63%		5	1.52	1.17–1.97	80%	
NOS scores	≤ 7 scores	3	1.32	1.00–1.75	72%	0.34	3	1.59	1.04–2.43	85%	0.25
	>7 scores	7	1.13	1.05–1.22	63%		6	1.23	1.08–1.39	74%	
Study design	Cross-section	2	1.18	0.97–1.44	32%	0.88	2	1.51	0.79–2.86	89%	0.66
	Cohort	8	1.17	1.08–1.28	74%		7	1.30	1.13–1.49	78%	
Sample size	<5,000	4	1.17	1.00–1.38	79%	0.67	4	1.33	1.16–1.51	78%	0.39
	≥5,000	6	1.19	1.08–1.30	61%		5	1.14	1.03–1.26	58%	
Population	General population	8	1.11	1.06–1.16	17%	<0.001	7	1.22	1.08–1.37	73%	0.001
	Diabetes mellitus population	2	1.86	1.49–2.32	0%		2	1.87	1.48–2.37	0%	
Adjusted for age	Yes	10	1.15	1.07–1.25	74%	NA	9	1.35	1.18–1.53	73%	NA
	No	0	NA	NA	NA		0	NA	NA	NA	
Adjusted for BMI	Yes	8	1.19	1.09–1.30	76%	0.34	7	1.32	1.15–1.51	70%	0.80
	No	2	1.09	0.93–1.28	0%		2	1.45	0.71–2.96	93%	
Adjusted for hypertension	Yes	7	1.21	1.09–1.35	72%	0.34	6	1.59	1.28–1.98	76%	0.002
	No	3	1.12	0.98–1.27	64%		3	1.10	1.01–1.18	29%	
Adjusted smoking status	Yes	7	1.14	1.09–1.19	0%	0.53	6	1.42	1.18–1.72	75%	0.21
	No	3	1.24	0.95–1.63	85%		3	1.20	0.99–1.45	83%	
Adjusted diabetes mellitus	Yes	6	1.11	1.06–1.17	38%	0.17	5	1.19	1.09–1.30	35%	0.15
	No	4	1.40	1.02–1.90	80%		4	1.64	1.06–2.51	91%	
Adjusted kidney function	Yes	3	1.14	1.07–1.20	0%	0.54	2	1.55	0.90–2.67	75%	0.62
	No	7	1.18	1.05–1.34	81%		7	1.34	1.15–1.56	76%	

*OR, odd ratio; NOS, Newcastle-Ottawa Scale; BMI, body mass index; CI, confidence interval; NA, not available*.

## Discussion

### Main Findings

Based on 10 eligible studies with 814,804 participants (415,779 men and 399,025 women), we further investigated the sex-specific association and found a positive linear dose-response relationship between SUA and AF in both men and women. For each 60 μmol/L (1 mg/dL) increase in SUA levels, the risk of AF increased by 15% in men and 35% in women. There was a borderline significant sex difference in the impact of SUA on the risk of AF, with the risk estimates being higher in women.

Two studies (30, 32) with diabetic patients were included in the analysis. In a subgroup of the population, we found that there was a stronger association between SUA and the risk of AF among the reports with diabetic populations, whether in men or women. Consistent with this trend, a study that included 245 patients showed that hyperuricemia increased the risk of AF by 341% in type 2 diabetes (24). Moreover, numerous studies have reported that SUA has a potential connection with diabetes (49). Insulin resistance in diabetic patients can increase SUA reabsorption. Similarly, diabetic nephropathy leads to a decrease in SUA excretion through the kidney (50–53). Other studies have shown that high levels of SUA are a risk factor for vascular disease in type 2 diabetes and insulin resistance (54). Therefore, the relationship between SUA and AF may be amplified in diabetic patients.

SUA predicts the development of hypertension (55), which is a well-known risk factor for AF. Evidence from a double-blinded, placebo-controlled study of prehypertensive adolescents showed that lowering SUA had a positive effect on blood pressure reduction (56). In our results, although adjustments for hypertension did not influence the positive associations between SUA and AF, an unexpectedly larger magnitude risk estimate was seen in women after adjustment for hypertension. To date, we have no logical explanation for these unexpected results. More studies will likely be needed to assess the effect of hypertension on the association between SUA and AF.

Interestingly, the category analysis of hyperuricemia vs. normal SUA showed patients with hyperuricemia were associated with increased risk of AF in men but not in women. No statistically significant difference among women (*P* = 0.14, Supplementary Figure S1) may due to the limited data.

### Sex-Related Difference

A sex-specific association between SUA and health was suggested by previous studies, such as stroke (57), left ventricular hypertrophy (58), metabolic syndrome (59), and coronary artery disease (60, 61). For example, the results from the First National Health and Nutrition Examination Survey (FNHANES) showed significant higher ischemic heart disease mortality, total cardiovascular mortality, and all-cause mortality in women with increasing SUA levels (62). In the context of AF, the results were also inconsistent. In several longitudinal studies, a significant association between high SUA and new-onset AF was observed in women but not in men (31, 35). However, other cohorts showed contrary results, with independent associations being significant in men but not in women (33, 38). In the present study, we showed that a high SUA was associated with a risk of AF in both men and women. However, there was a borderline significant sex difference in the impact of SUA on the risk of AF, with risk estimates being higher in women. This trend was consistent in non-linear analysis, showing a likelihood of a steeper increase in women with increasing SUA. Moreover, an analysis based on a Chronic Condition Data Warehouse showed that the reduced risk estimate of incident AF with allopurinol use was more prominent in women (63), which reinforced our results in the context of sex differences. The mechanism underlying SUA and AF has been discussed previously (28). The mechanism underlying different sex-based patterns remains unclear and may be due to differences in sex hormones. In general, estrogen is well known to have a heart-protecting effect, such as reducing the number of uric acid transporters in the kidney. However, it has been reported that estrogen can induce increased expression of urate transporters in female patients (64, 65). SUA enters cardiomyocytes through urate transporters, which increases the expression of kvl.5 protein, thus shortening the action potential duration and leading to the occurrence and progression of AF (66, 67). In addition, variations in SUA levels between men and women are thought to be linked to increased renal urate reabsorption in response to testosterone (68). Moreover, in certain large cohorts, an association between low levels of circulating testosterone and incident AF has been discovered (69, 70). Decreased testosterone levels have been shown in animal models to promote proarrhythmic alterations in calcium management, which might increase the risk of AF (71, 72). Further studies are warranted to elucidate the underlying biological mechanisms of sex-specific patterns.

The occurrence of AF increases with age. SUA levels in postmenopausal women are increased due to metabolic changes caused by menopause (73), which might contribute to the higher incidence of AF in older women. In contrast, in men, flat or slightly declining SUA levels are observed with aging (74). This age-related change in SUA might influence the sex differences associated with the AF risk. However, in the present study, the mean age of the women ranged from 38 to 57 years in the majority of studies, and the mean age of all patients in the included studies was under 65 years. The subgroup of elderly individuals was not available, which precluded us from further assessing the sex difference stratified by elderly individuals. Notably, a trend of sex difference persisted in the SUA-associated AF after adjustment for age (OR = 1.35 for women and OR = 1.15 for men), although with a non-significant *P*-value (0.1). Therefore, our results do not seem to support the hypothesis that increasing age accounts for the sex-based difference in the context of SUA and AF.

### Clinical Implications

Considering the proven association between high SUA and an increased risk of AF, SUA-lowering therapy may be a therapeutic target for reducing the risk of AF in the future. A retrospective, single-center study of 603 patients with heart failure showed that the use of allopurinol reduced the risk of AF by 13% (75). In a retrospective cohort study including 9,244 elderly patients, Singh et al. reported that patients receiving allopurinol had a lower incidence of AF and that longer usage of allopurinol was associated with a lower risk of AF, heart failure mortality and myocardial infarction (63). Consistently, a recent meta-analysis suggested that allopurinol treatment of hyperuricemia was associated with improved endothelial function, which indicated that allopurinol might have a cardioprotective effect in addition to lowering the SUA levels (76), especially for those with cardiovascular diseases, such as diabetes. However, febuxostat might not be ideal because it increases the risk of cardiac and all-cause death compared with allopurinol (77). Thus, the Food and Drug Administration has recently issued a safety alert for febuxostat, which increases the risk of cardiovascular events in patients (78). Considering that merely reducing serum urate with a drug such as febuxostat has not been proven to benefit cardiac health, the benefits of allopurinol in lowering the incidence of AF may arise from a mechanism of action other than lowering urate. Moreover, we also found that the cutoff value of SUA was higher in women than in men for the association with AF. Additional studies are required to confirm this sex difference, and trials are also needed to clarify the optimal SUA levels and the safety of various SUA-lowering drugs for the future prevention of AF.

As we know, SUA level was regulated by kidney function. Evidence from recent studies showed renal dysfunction could impair the excretion of uric acid and contribute to a higher prevalence of hyperuricemia in the elderly. We performed a subgroup analysis stratified by adjustment for renal function. A positive association between SUA and the risk of AF among men and women persisted and there was little evidence of heterogeneity. Therefore, the association between SUA and risk of AF might be independent of kidney function.

### Strengths and Limitation

Our results appear to agree with previous meta-analyses (26–28), which suggested that high SUA is associated with an increased prevalence and risk of AF. More importantly, our meta-analysis extended these studies and had three important strengths. First, to the best of our knowledge, this is the first dose-response meta-analysis that explored the sex-specific associations of SUA with the risk of AF. Second, all of the included studies adjusted for clinical confounding factors, indicating that our results were relatively stable. Third, the strong associations between SUA and the risk of AF among men and women persisted in all subgroup analyses and were reliable in the sensitivity analyses, suggesting that elevated SUA may be an independent risk factor for AF in both men and women.

However, there are several limitations to the present systematic review and meta-analysis that need to be discussed. First, all of the included studies were observational studies. Hence, additional randomized, controlled trials are warranted to properly validate the associations of the SUA levels with the risk of AF in both men and women. Second, a high degree of heterogeneity was observed in our results. This was not surprising because of variations in the characteristics of the study populations and study designs. Third, although the trim-and-fill method was applied to address the problem of publication bias, such a test could have limited power in the setting of relatively few studies (79). Thus, the risk estimates may be overstated due to potential studies with null results.

## Conclusions

The findings of this study suggest that there is a significant linear relationship between SUA and the risk of AF among men and women, with a higher risk estimate in women. Additional trials are required to assess the effect of lowering SUA therapy on the AF incidence.

## Data Availability Statement

The original contributions presented in the study are included in the article/Supplementary Material, further inquiries can be directed to the corresponding author/s.

## Author Contributions

XL and KM were responsible for the entire project and revised the draft. JX and WS performed the study selection, data extraction, statistical analysis, and interpreting the data. WS drafted the first version of the manuscript. All authors participated in the interpretation of the results and prepared the final version of the manuscript.

## Funding

This work was supported by the National Natural Science Foundation of China (PY, 81760050 and 81760048; XL, 82100347), China Postdoctoral Science Foundation (XL, 2021M703724), the Jiangxi Provincial Natural Science Foundation for Youth Scientific Research (PY, 20192ACBL21037), and the Jiangxi Provincial Key Laboratory of Cardiovascular Diseases of Traditional Chinese Medicine (20212BCD42010).

## Conflict of Interest

The authors declare that the research was conducted in the absence of any commercial or financial relationships that could be construed as a potential conflict of interest.

## Publisher's Note

All claims expressed in this article are solely those of the authors and do not necessarily represent those of their affiliated organizations, or those of the publisher, the editors and the reviewers. Any product that may be evaluated in this article, or claim that may be made by its manufacturer, is not guaranteed or endorsed by the publisher.

## Abbreviations

AF: atrial fibrillation
SUA: serum uric acid
XO: xanthine oxidoreductase
OR: odds ratio
RR: risk ratio
HR: hazard ratio
NOS: Newcastle-Ottawa Scale
BMI: body mass index.
