# Supplementary material for: Hyperuricemia Is Associated With the Risk of Atrial Fibrillation Independent of Sex: A Dose-Response Meta-Analysis
Source: Front Cardiovasc Med. 2022 Apr 7;9:865036. doi: 10.3389/fcvm.2022.865036 (PMC9021846; doi:10.3389/fcvm.2022.865036)
Supplement: Supplementary file 1 [file Data_Sheet_1.docx]

**Hyperuricemia is associated with the risk of atrial fibrillation independent of sex: A dose-response meta-analysis**

**Running title:** uric acid and atrial fibrillation

**Supplementary Table 1. PRISMA Checklist.**

| **Section/topic** | **#** | **Checklist item** | **Reported on page #** |
| --- | --- | --- | --- |
| **TITLE** | | |  |
| Title | 1 | Identify the report as a systematic review, meta-analysis, or both. | 1 |
| **ABSTRACT** | | |  |
| Structured summary | 2 | Provide a structured summary including, as applicable: background; objectives; data sources; study eligibility criteria, participants, and interventions; study appraisal and synthesis methods; results; limitations; conclusions and implications of key findings; systematic review registration number. | 3 |
| **INTRODUCTION** | | |  |
| Rationale | 3 | Describe the rationale for the review in the context of what is already known. | 4 |
| Objectives | 4 | Provide an explicit statement of questions being addressed with reference to participants, interventions, comparisons, outcomes, and study design (PICOS). | 4 |
| **METHODS** | | |  |
| Protocol and registration | 5 | Indicate if a review protocol exists, if and where it can be accessed (e.g., Web address), and, if available, provide registration information including registration number. | 5 |
| Eligibility criteria | 6 | Specify study characteristics (e.g., PICOS, length of follow-up) and report characteristics (e.g., years considered, language, publication status) used as criteria for eligibility, giving rationale. | 6 |
| Information sources | 7 | Describe all information sources (e.g., databases with dates of coverage, contact with study authors to identify additional studies) in the search and date last searched. | 5 |
| Search | 8 | Present full electronic search strategy for at least one database, including any limits used, such that it could be repeated. | 6 |
| Study selection | 9 | State the process for selecting studies (i.e., screening, eligibility, included in systematic review, and, if applicable, included in the meta-analysis). | 6 |
| Data collection process | 10 | Describe method of data extraction from reports (e.g., piloted forms, independently, in duplicate) and any processes for obtaining and confirming data from investigators. | 6 |
| Data items | 11 | List and define all variables for which data were sought (e.g., PICOS, funding sources) and any assumptions and simplifications made. | 6 |
| Risk of bias in individual studies | 12 | Describe methods used for assessing risk of bias of individual studies (including specification of whether this was done at the study or outcome level), and how this information is to be used in any data synthesis. | 7 |
| Summary measures | 13 | State the principal summary measures (e.g., risk ratio, difference in means). | 7 |
| Synthesis of results | 14 | Describe the methods of handling data and combining results of studies, if done, including measures of consistency (e.g., I^2^) for each meta-analysis. | 7 |
| Risk of bias across studies | 15 | Specify any assessment of risk of bias that may affect the cumulative evidence (e.g., publication bias, selective reporting within studies). | 7 |
| Additional analyses | 16 | Describe methods of additional analyses (e.g., sensitivity or subgroup analyses, meta-regression), if done, indicating which were pre-specified. | 7 |
| **RESULTS** | | |  |
| Study selection | 17 | Give numbers of studies screened, assessed for eligibility, and included in the review, with reasons for exclusions at each stage, ideally with a flow diagram. | 8 |
| Study characteristics | 18 | For each study, present characteristics for which data were extracted (e.g., study size, PICOS, follow-up period) and provide the citations. | 8 |
| Risk of bias within studies | 19 | Present data on risk of bias of each study and, if available, any outcome level assessment (see item 12). | 10 |
| Results of individual studies | 20 | For all outcomes considered (benefits or harms), present, for each study: (a) simple summary data for each intervention group (b) effect estimates and confidence intervals, ideally with a forest plot. | 9 |
| Synthesis of results | 21 | Present results of each meta-analysis done, including confidence intervals and measures of consistency. | 10 |
| Risk of bias across studies | 22 | Present results of any assessment of risk of bias across studies (see Item 15). | 10 |
| Additional analysis | 23 | Give results of additional analyses, if done (e.g., sensitivity or subgroup analyses, meta-regression [see Item 16]). | 11 |
| **DISCUSSION** | | |  |
| Summary of evidence | 24 | Summarize the main findings including the strength of evidence for each main outcome; consider their relevance to key groups (e.g., healthcare providers, users, and policy makers). | 11-15 |
| Limitations | 25 | Discuss limitations at study and outcome level (e.g., risk of bias), and at review-level (e.g., incomplete retrieval of identified research, reporting bias). | 15 |
| Conclusions | 26 | Provide a general interpretation of the results in the context of other evidence, and implications for future research. | 16 |
| **FUNDING** | | |  |
| Funding | 27 | Describe sources of funding for the systematic review and other support (e.g., supply of data); role of funders for the systematic review. | 17 |

*From:*  Moher D, Liberati A, Tetzlaff J, Altman DG, The PRISMA Group (2009). Preferred Reporting Items for Systematic Reviews and Meta-Analyses: The PRISMA Statement. PLoS Med 6(7): e1000097. doi:10.1371/journal.pmed1000097

For more information, visit: **www.prisma-statement.org**.

**Table S2.** Studies excluded (n=21) with reasons

| **Studies excluded** | **Reasons** |
| --- | --- |
| Chao, 2013(1) | Without the target exposure: one episode of gout attack |
| Chao, 2014(2) | Without the target outcome: ischemic stroke |
| Lliesiu,2010(3) | This is a review |
| Guo, 2014(4) | Do not meet the inclusion: univariate analysis |
| Lin,2019(5) | Not the target exposure: serum urate |
| Stanhope,2018(6) | This is a review |
| Kim, 2015(7) | Not the target outcome: composite cardiovascular diseases |
| Hong,2020(8) | Without the target sex-specific outcomes |
| Kwon,2018(9) | Without the target sex-specific outcomes |
| Letsas,2013(10) | Without the target sex-specific outcomes |
| He,2013(11) | Without the target sex-specific outcomes |
| Kim,2015(7) | Without the target sex-specific outcomes |
| Chuang,2014(12) | Without the target sex-specific outcomes |
| Canpolat,2014 | Without the target sex-specific outcomes |
| Li, 2016(13) | This is a meta-analysis |
| Correa,2021(14) | Without the target sex-specific outcomes |
| Kobayashi,2021(15) | Without the target sex-specific outcomes |
| Ono, 2016(16) | This is an editorial |
| Singh,2017(17) | Without the target exposure: allopurinol |
| Tamariz,2014(18) | This is a meta-analysis |
| Singh,2018(19) | Without the target exposure: gout attack |
| Kuo,2016(20) | Without the target exposure: gout attack |
| Zhu, 2020(21) | Sample size<200 |

1. Chao TF, Hung CL, Chen SJ, Wang KL, Chen TJ, Lin YJ, et al. The association between hyperuricemia, left atrial size and new-onset atrial fibrillation. Int J Cardiol. 2013;168(4):4027-32. doi: 10.1016/j.ijcard.2013.06.067. PubMed PMID: 23871344.

2. Chao TF, Liu CJ, Chen SJ, Wang KL, Lin YJ, Chang SL, et al. Hyperuricemia and the risk of ischemic stroke in patients with atrial fibrillation--could it refine clinical risk stratification in AF? Int J Cardiol. 2014;170(3):344-9. doi: 10.1016/j.ijcard.2013.11.011. PubMed PMID: 24290426.

3. Iliesiu A, Campeanu A, Dusceac D. Serum uric acid and cardiovascular disease. Mædica. 2010;5(3):186.

4. Guo X, Zhang S, Yan X, Chen Y, Yu R, Long D, et al. Postablation neutrophil/lymphocyte ratio correlates with arrhythmia recurrence after catheter ablation of lone atrial fibrillation. Chinese medical journal. 2014;127(6):1033-8.

5. Lin S, Cheng J, Cui L, Gurol ME, Bhatt DL, Fonarow GC, et al. Cohort Study of Repeated Measurements of Serum Urate and Risk of Incident Atrial Fibrillation. J Am Heart Assoc. 2019;8(13):e012020. doi: 10.1161/JAHA.119.012020. PubMed PMID: 31213103; PubMed Central PMCID: PMCPMC6662349.

6. Stanhope KL, Goran MI, Bosy‐Westphal A, King JC, Schmidt LA, Schwarz JM, et al. Pathways and mechanisms linking dietary components to cardiometabolic disease: Thinking beyond calories. Obesity reviews. 2018;19(9):1205-35.

7. Kim SC, Schneeweiss S, Choudhry N, Liu J, Glynn RJ, Solomon DH. Effects of xanthine oxidase inhibitors on cardiovascular disease in patients with gout: a cohort study. Am J Med. 2015;128(6):653 e7- e16. doi: 10.1016/j.amjmed.2015.01.013. PubMed PMID: 25660249; PubMed Central PMCID: PMCPMC4442710.

8. Hong M, Park JW, Yang PS, Hwang I, Kim TH, Yu HT, et al. A mendelian randomization analysis: The causal association between serum uric acid and atrial fibrillation. European Journal of Clinical Investigation. 2020;50(10):e13300.

9. Kwon CH, Lee SH, Lee JY, Ryu S, Sung KC. Uric Acid and Risk of Atrial Fibrillation in the Korean General Population. Circ J. 2018;82(11):2728-35. doi: 10.1253/circj.CJ-18-0748. PubMed PMID: 30232315.

10. Letsas KP, Siklody CH, Korantzopoulos P, Weber R, Burkle G, Mihas CC, et al. The impact of body mass index on the efficacy and safety of catheter ablation of atrial fibrillation. Int J Cardiol. 2013;164(1):94-8. doi: 10.1016/j.ijcard.2011.06.092. PubMed PMID: 21726910.

11. He XN, Li SN, Zhan JL, Xie SL, Zhang ZJ, Dong JZ, et al. Serum uric acid levels correlate with recurrence of paroxysmal atrial fibrillation after catheter ablation. Chin Med J (Engl). 2013;126(5):860-4. doi: 10.3760/cma.j.issn.0366-6999.20122154. PubMed PMID: 23489791.

12. Chuang SY, Wu CC, Hsu PF, Chia-Yu Chen R, Liu WL, Hsu YY, et al. Hyperuricemia and incident atrial fibrillation in a normotensive elderly population in Taiwan. Nutr Metab Cardiovasc Dis. 2014;24(9):1020-6. doi: 10.1016/j.numecd.2014.03.012. PubMed PMID: 24993310.

13. Li M, Hu X, Fan Y, Li K, Zhang X, Hou W, et al. Hyperuricemia and the risk for coronary heart disease morbidity and mortality a systematic review and dose-response meta-analysis. Sci Rep. 2016;6:19520. doi: 10.1038/srep19520. PubMed PMID: 26814153; PubMed Central PMCID: PMCPMC4728388.

14. Correa S, Curtis KA, Waikar SS, Mc Causland FR. Serum Myeloperoxidase, Uric Acid, and the Risk of Atrial Fibrillation in Chronic Kidney Disease. Circ Arrhythm Electrophysiol. 2021;14(4):e009483. doi: 10.1161/CIRCEP.120.009483. PubMed PMID: 33858180.

15. Kobayashi T, Kokubo Y, Higashiyama A, Watanabe M, Nakao YM, Kamakura S, et al. Uric acid and incident atrial fibrillation of 14 years population‐based cohort study: The Suita Study. Journal of arrhythmia. 2021;37(5):1215-9.

16. Ono K. How Is Uric Acid Related to Atrial Fibrillation? Circ J. 2019;83(4):705-6. doi: 10.1253/circj.CJ-19-0134. PubMed PMID: 30814432.

17. Singh JA, Yu S. Allopurinol and the risk of atrial fibrillation in the elderly: a study using Medicare data. Ann Rheum Dis. 2017;76(1):72-8. doi: 10.1136/annrheumdis-2015-209008. PubMed PMID: 27165177.

18. Tamariz L, Agarwal S, Soliman EZ, Chamberlain AM, Prineas R, Folsom AR, et al. Association of serum uric acid with incident atrial fibrillation (from the Atherosclerosis Risk in Communities [ARIC] study). Am J Cardiol. 2011;108(9):1272-6. doi: 10.1016/j.amjcard.2011.06.043. PubMed PMID: 21855838; PubMed Central PMCID: PMCPMC3404126.

19. Singh JA, Cleveland JD. Gout and the risk of incident atrial fibrillation in older adults: a study of US Medicare data. RMD Open. 2018;4(2):e000712. doi: 10.1136/rmdopen-2018-000712. PubMed PMID: 30018808; PubMed Central PMCID: PMCPMC6045725.

20. Kuo Y-J, Tsai T-H, Chang H-P, Chua S, Chung S-Y, Yang C-H, et al. The risk of atrial fibrillation in patients with gout: a nationwide population-based study. Scientific Reports. 2016;6(1). doi: 10.1038/srep32220.

21. Zhu T, Wang Z, Wang S, Hu W, Chen H, Xie J, et al. Association between Serum Adiponectin and Atrial Fibrillation: A Case-Control Study Stratified by Age and Gender. Cardiol Res Pract. 2021;2021:6633948. doi: 10.1155/2021/6633948. PubMed PMID: 33628489; PubMed Central PMCID: PMCPMC7889381.

**Supplementary Table 3. Joanna Briggs Institute critical appraisal checklist applied for included studies.**

| Study | Sample was representative? | Participants appropriately recruited? | Sample size was adequate? | Study subjects and the setting described | Data analysis conducted | Objective, standard criteria, reliably used? | Appropriate statistical analysis used? | Confounding factors/ subgroups/ differences identified and accounted? | Subpopulations identified using objective criteria |
| --- | --- | --- | --- | --- | --- | --- | --- | --- | --- |
| Chen, 2017 | Yes | Yes | Yes | Yes | Yes | Yes | Yes | Yes | Yes |
| Sun, 2015 | Yes | Yes | Yes | Yes | Yes | Yes | Yes | Yes | Yes |

**Supplementary Table 4. Quality assessment of the included studies by Newcastle–Ottawa scale.**

| Author  (Publication Year) | Newcastle-Ottawa Scale | | | | | | | | | |
| --- | --- | --- | --- | --- | --- | --- | --- | --- | --- | --- |
|  | Selection | | | Comparability | | | Outcome | | | Total |
|  | a | b | c | d | e | f | g | h | i |  |
| Kwon, 2018 | 1 | 1 | 1 | 1 | 1 | 1 | 1 | 1 | 1 | 9 |
| Nyrnes, 2014 | 1 | 1 | 1 | 1 | 1 | 1 | 1 | 1 | 1 | 9 |
| Peters, 2018 | 1 | 1 | 1 | 1 | 1 | 0 | 1 | 1 | 1 | 8 |
| Valbusa, 2013 | 1 | 1 | 1 | 1 | 1 | 1 | 1 | 1 | 1 | 9 |
| Tamariz, 2011 | 1 | 1 | 1 | 1 | 1 | 1 | 1 | 1 | 1 | 9 |
| Kawasoe, 2018 | 1 | 1 | 1 | 1 | 1 | 1 | 1 | 0 | 1 | 8 |
| Ding, 2015 | 1 | 1 | 1 | 1 | 1 | 1 | 1 | 0 | 0 | 7 |
| Seki, 2021 | 1 | 1 | 1 | 1 | 1 | 1 | 1 | 1 | 1 | 9 |

1. Representativeness of the exposed cohort.
2. Selection of the non-exposed cohort.
3. Ascertainment of exposure.
4. Demonstration that outcome of interest was not present at start of study.
5. Comparability of cohorts on the basis of the design or analysis (adjusted for age).
6. Comparability of cohorts on the basis of the design or analysis (adjusted for any other factor).
7. Assessment of outcome.
8. Was follow-up long enough for outcomes to occur. (>5 years for new on-set, 1 years for AF recurrence).
9. Adequacy of follow-up of cohorts.

**Supplementary Table 5. Sensitivity analyses of the associations between serum uric acid levels and atrial fibrillation in men and women.**

|  | Men | | | | Women | | | |
| --- | --- | --- | --- | --- | --- | --- | --- | --- |
|  | N | OR (95% CI) | P | I^2^ (%) | N | OR (95% CI) | P | I^2^ (%) |
| Categorical analysis of SUA and AF, highest-versus-lowest |  |  |  |  |  |  |  |  |
| Primary outcomes | 6 | 1.42 (1.18-1.71) | <0.001 | 34 | 6 | 2.02 (1.29-3.16) | 0.002 | 70 |
| Excluded studies using ICD to detect AF | 5 | 1.49 (1.15-1.94) | 0.003 | 46 | 5 | 2.37 (1.42-3.95) | <0.001 | 64 |
| Dose-response association between SUA and risk of AF |  |  |  |  |  |  |  |  |
| Primary outcomes | 10 | 1.15 (1.07-1.25) | <0.001 | 74 | 9 | 1.35 (1.18-1.53) | <0.001 | 73 |
| Excluded studies using ICD to detect AF | 9 | 1.17 (1.07-1.28) | 0.001 | 77 | 9 | 1.35 (1.18-1.53) | <0.001 | 73 |

N: number of studies; OR: odd ratio; CI: confidence interval; SUA: serum uric acid; AF: atrial fibrillation; ICD: implantable cardioverter defibrillator.

**Figure S1. Forest plot for the risk of AF with hyperuricemia versus the normal serum uric acid among men (a) and women (b).**


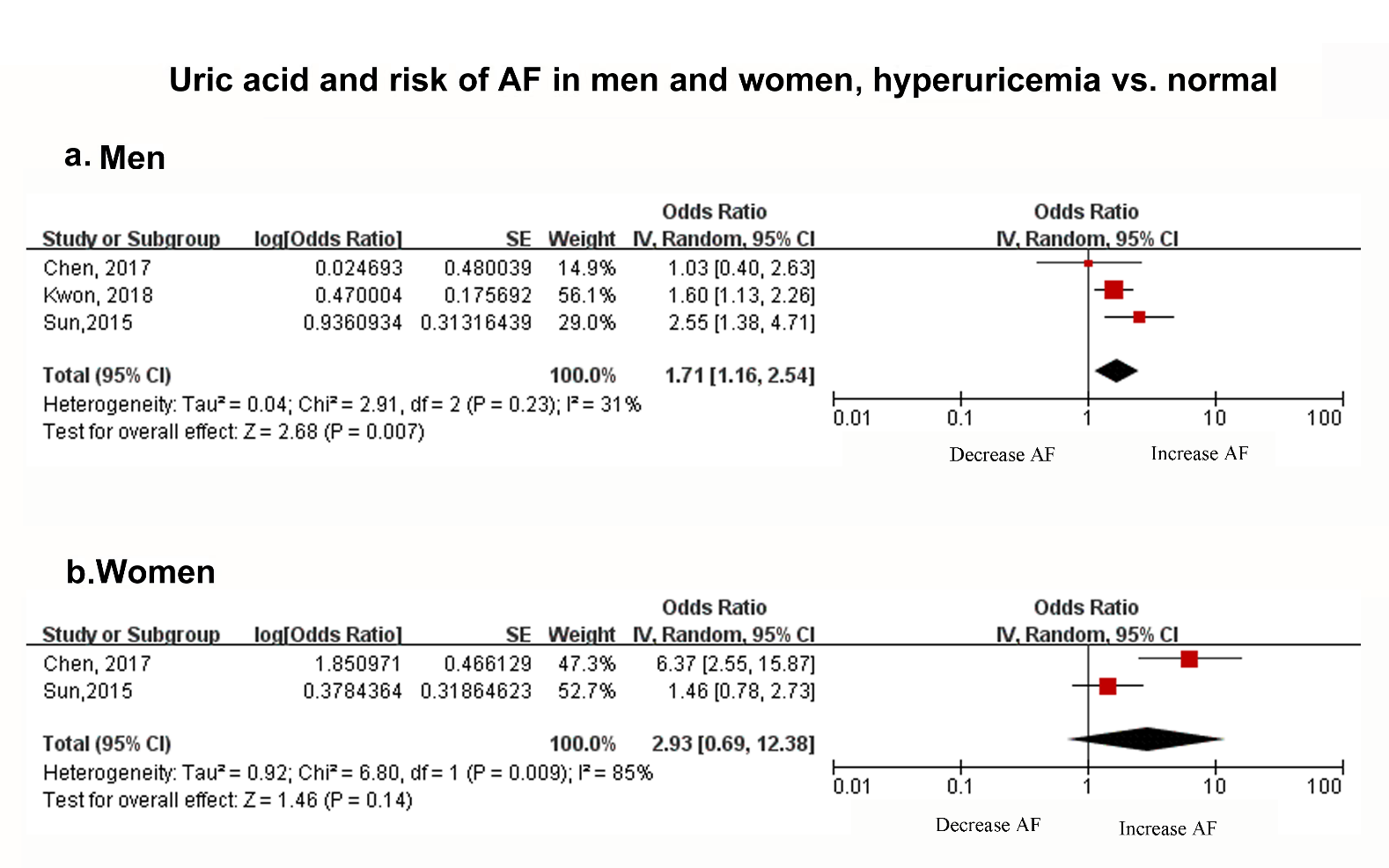
**Note: AF=atrial fibrillation**

**Figure S2. Publication bias detected by funnel plot, Egger’s test, and Begg’s test for the association between serum uric acid levels and atrial fibrillation.**

**A. Category analysis among men:** upper: funnel plot, middle: Egger’s test (p=0.294), lower: Begg’s test (p=0.452). **B. Category analysis among women:** upper: funnel plot, middle: Egger’s test (p=0.023), lower: Begg’s test (p=0.260). **C. Dose-response analysis among men:** upper: funnel plot, middle: Egger’s test (p=0.014), lower: Begg’s test (p=0.020). **D. Dose-response analysis among women:** upper: funnel plot, middle: Egger’s test (p=0.004), lower: Begg’s test (p=0.118).

**
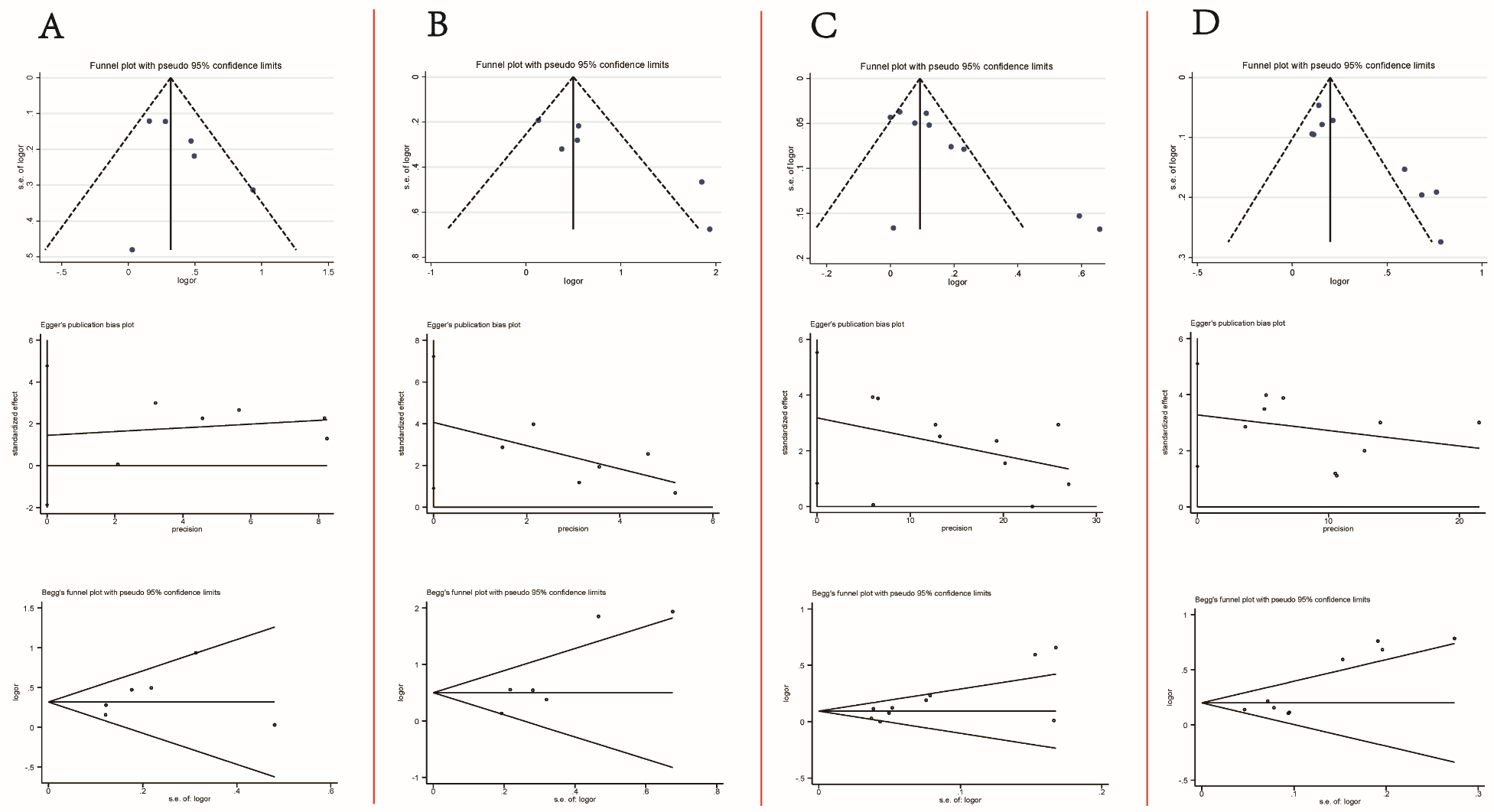
**

**Figure S3. Publication bias detected by the trim-and-fill method for the association between serum uric acid levels and atrial fibrillation.**


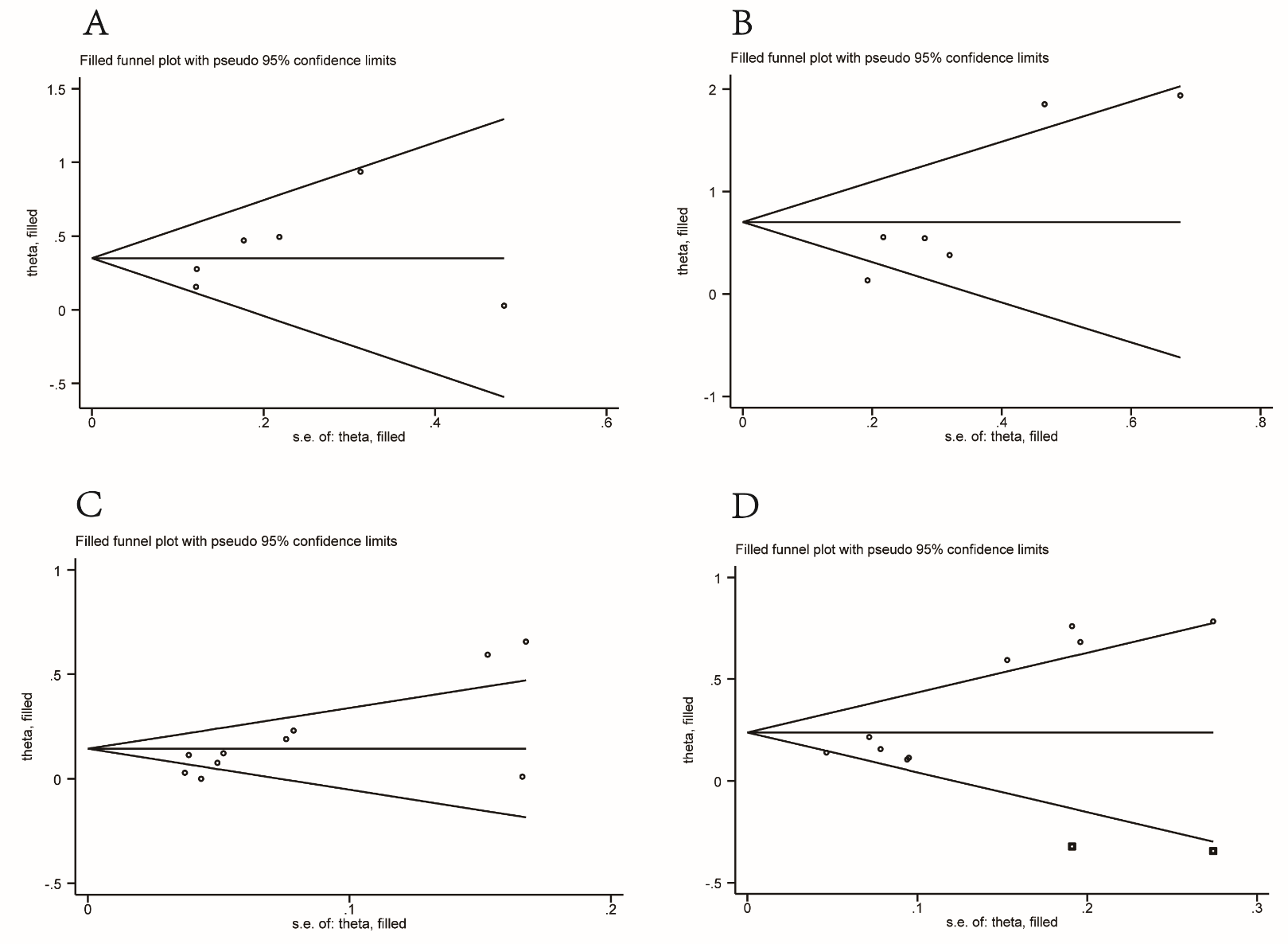
**A. Category analysis among men. B. Category analysis among women. C. Dose-response analysis among men. D. Dose-response analysis among women.**

**Figure S4. Sensitivity analysis of included studies that reported the association between serum uric acid levels and atrial fibrillation, by omitting each study at once.**

**
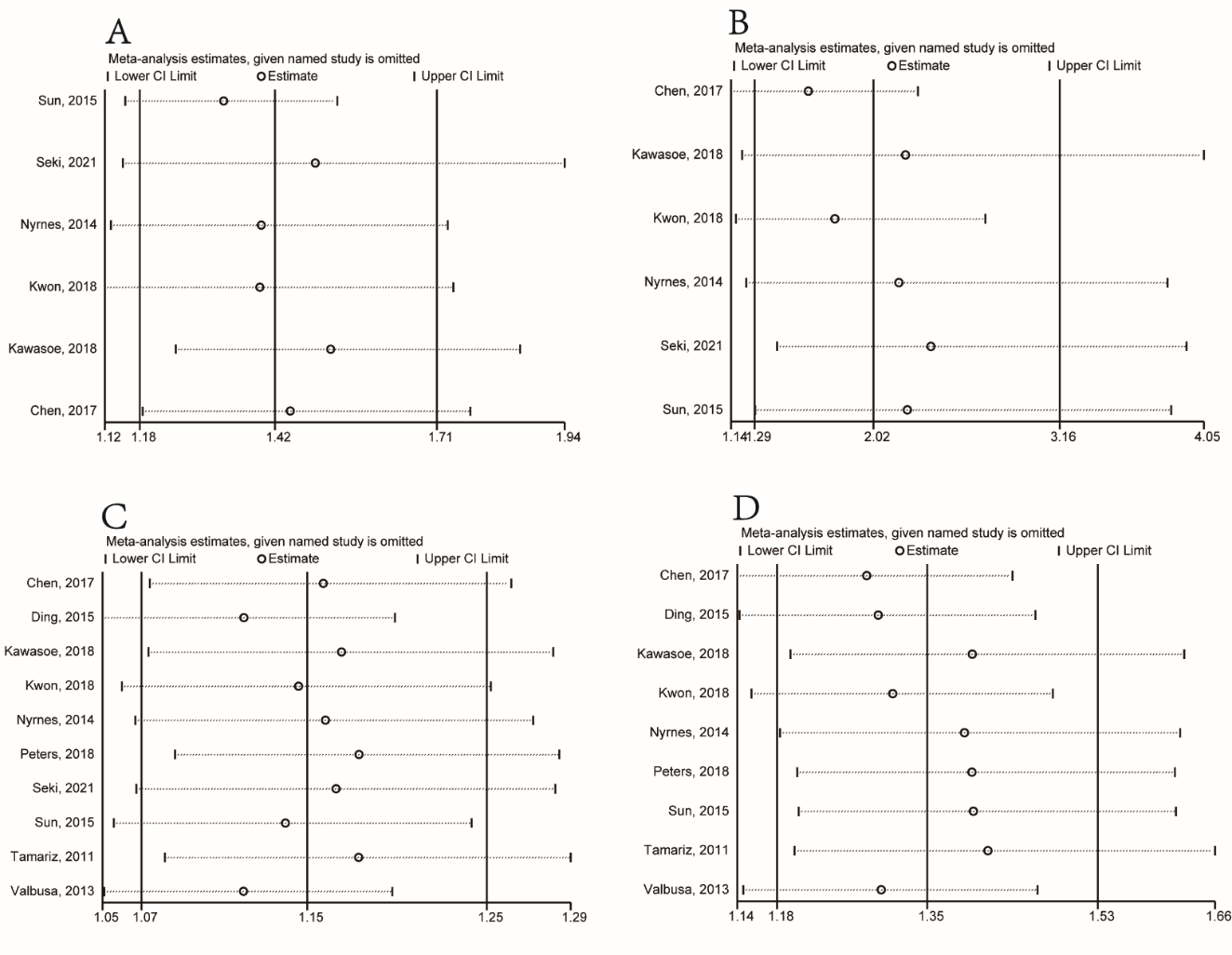
A. Category analysis among men. B. Category analysis among women. C. Dose-response analysis among men. D. Dose-response analysis among women.**

**Figure S4. Sensitivity analysis of included studies that reported the association between serum uric acid levels and atrial fibrillation, by omitting studies without kidney function adjustments.**
